# Canakinumab in the routinary clinical practice in cryopyrin-associated periodic syndromes (CAPS): one year of follow-up

**DOI:** 10.1186/1546-0096-9-S1-P22

**Published:** 2011-09-14

**Authors:** R Caorsi, L Lepore, F Zulian, M Alessio, A Stabile, M Finetti, A Martini, M Gattorno

**Affiliations:** 1 UO Pediatria II, G. Gaslini Institute and Department of Pediatrics, University of Genoa, Genova, Italy; 2 IRCCS Burlo Garofalo, Dipartimento di Pediatria, University of Trieste, Trieste, Italy; 3 Dipartimento A.I. di Pediatria, University of Padua, Padova, Italy; 4 Department of Pediatrics, Federico II Hospital, Napoli, Italy; 5 Department of Paediatrics, A. Gemelli Hospital, University of Rome, Italy

## Background

No clear information on the optimal dosage of Canakinumab in CAPS is available.

## Aim

To analyse the modification of dosage schedule of Canakinumab in CAPS in 12 months of routinely clinical practice.

## Methods

12 patients (9 children and 3 adults) with Muckle-Wells syndrome (3), MWS/CINCA overlap (3) and CINCA (6) were analyzed. Patients were previously enrolled in the CACZ885D2306 trial and studied for the following 12 months.

## Results

At baseline, 7 patients were treated with the initial dosage of 2 mg/kg (or 150 mg, if > 40 Kg) every 8 weeks. In 5 patients (2 MWS/CINCA overlap, 5 CINCA) the dosage was 4 mg/kg (or 300 mg) every 8 weeks.

During the following 12 months modification of dosage of frequency was performed in 7/12 patients. The 5 patients at higher dosage during the CACZ885D2306 study needed to increase the frequency of administration with a mean frequency of 6 weeks (range 4-8). The mean reason was the presence of mild clinical manifestation and/or persistent elevation of acute phase reactants. In one of these patients the therapy was subsequently discontinued due to persistent disease activity. An increased frequency (6 and 7 weeks) was also performed in 1 MWS and 1 CINCA patient, respectively.

In 5 patients the treatment was not modified being effective in the control of the disease.

## Conclusions

This study confirms the efficacy of Canakinumab in CAPS. However, pediatric patients and those with a more severe phenotype require higher and more frequent dosage than previously described.

